# Intravoxel incoherent motion and enhanced T2*-weighted angiography for preoperative prediction of microvascular invasion in hepatocellular carcinoma

**DOI:** 10.3389/fonc.2024.1389769

**Published:** 2024-08-09

**Authors:** Xue Ren, Ying Zhao, Nan Wang, Jiahui Liu, Shuo Zhang, Mingrui Zhuang, Hongkai Wang, Jixiang Wang, Yindi Zhang, Qingwei Song, Ailian Liu

**Affiliations:** ^1^ Department of Radiology, The First Affiliated Hospital of Dalian Medical University, Dalian, China; ^2^ College of Medical Imaging, Dalian Medical University, Dalian, China; ^3^ College of Biomedical Engineering, Dalian University of Technology, Dalian, China

**Keywords:** hepatocellular carcinoma, microvascular invasion, intravoxel incoherent motion, enhanced T2*-weighted angiography, intratumoral susceptibility signal

## Abstract

**Objective:**

To investigate the value of the combined application of intravoxel incoherent motion (IVIM) and enhanced T2*-weighted angiography (ESWAN) for preoperative prediction of microvascular invasion (MVI) in hepatocellular carcinoma (HCC).

**Materials and methods:**

76 patients with pathologically confirmed HCC were retrospectively enrolled and divided into the MVI-positive group (n=26) and MVI-negative group (n=50). Conventional MRI, IVIM, and ESWAN sequences were performed. Three region of interests (ROIs) were placed on the maximum axial slice of the lesion on D, D*, and f maps derived from IVIM sequence, and R2* map derived from ESWAN sequence, and intratumoral susceptibility signal (ITSS) from the phase map derived from ESWAN sequence was also automatically measured. Receiver operating characteristic (ROC) curves were drawn to evaluate the ability for predicting MVI. Univariate and multivariate logistic regression were used to screen independent risk predictors in clinical and imaging information. The Delong’s test was used to compare the differences between the area under curves (AUCs).

**Results:**

The D and D* values of MVI-negative group were significantly higher than those of MVI-positive group (*P*=0.038, and *P*=0.023), which in MVI-negative group were 0.892×10^-3^ (0.760×10^-3^, 1.303×10^-3^) mm^2^/s and 0.055 (0.025, 0.100) mm^2^/s, and in MVI-positive group were 0.591×10^-3^ (0.372×10^-3^, 0.824×10^-3^) mm^2^/s and 0.028 (0.006, 0.050)mm^2^/s, respectively. The R2* and ITSS values of MVI-negative group were significantly lower than those of MVI-positive group (*P*=0.034, and *P*=0.005), which in MVI-negative group were 29.290 (23.117, 35.228) Hz and 0.146 (0.086, 0.236), and in MVI-positive group were 43.696 (34.914, 58.083) Hz and 0.199 (0.155, 0.245), respectively. After univariate and multivariate analyses, only AFP (odds ratio, 0.183; 95% CI, 0.041–0.823; *P* = 0.027) was the independent risk factor for predicting the status of MVI. The AUCs of AFP, D, D*, R2*, and ITSS for prediction of MVI were 0.652, 0.739, 0.707, 0.798, and 0.657, respectively. The AUCs of IVIM (D+D*), ESWAN (R2*+ITSS), and combination (D+D*+R2*+ITSS) for predicting MVI were 0.772, 0.800, and, 0.855, respectively. When IVIM combined with ESWAN, the performance was improved with a sensitivity of 73.1% and a specificity of 92.0% (cut-off value: 0.502) and the AUC was significantly higher than AFP (*P*=0.001), D (*P*=0.038), D* (*P*=0.023), R2* (*P*=0.034), and ITSS (*P*=0.005).

**Conclusion:**

The IVIM and ESWAN parameters showed good efficacy in prediction of MVI in patients with HCC. The combination of IVIM and ESWAN may be useful for noninvasive prediction of MVI before clinical operation.

## 1 Introduction

Hepatocellular carcinoma (HCC) is the sixth most common cancer and the fourth leading cause of cancer-related deaths in the world (1). Liver resection, transplantation, and ablation are recommended as the most effective treatments of early stage HCC, and surgical resection is considered to be the best method for patients with well-preserved liver function (2). However, numerous clinical studies have indicated that the 5-year recurrence rate of HCC after surgery is as high as 70% (3).

Scholars have found that microvascular invasion (MVI) is the independent risk factor of early recurrence in HCC (4). In the presence of MVI, tumor cells can spread and metastasize in the liver, leading a portal vein tumor thrombus or distant metastasis (5). A previous study (6) reported that the 1-year recurrence rates for patients with and without MVI after curative resection for HCC were 23.3% and 7.5%, respectively. For HCC with MVI, anatomical resection is more recommended than non-anatomical resection (7), and resection with wide margin (margin ≥ 1 cm) is more recommended than narrow margin (8). Therefore, preoperative prediction of MVI is crucial for decision-making of surgical resection for patients with HCC (9).

MVI is diagnosed only by histopathological examination after operation or biopsy. However, the heterogeneity of HCC may lead to false-negative results (10). In addition, invasiveness of operation and time consumption also limit its usefulness on clinical-decision making. Magnetic resonance imaging (MRI) can effectively diagnose liver tumors, and the information reflecting the fine structure of organs in MRI can be mined. MRI-based radiological features can be used to detect MVI (11). Scholars found that larger tumor size (12), rim arterial enhancement (13), arterial peritumoral enhancement (14), and nonsmooth tumor margin (15) were significantly associated with MVI. However, the review of medical images rely on subjective experience, which lead to the inconsistency of the results (16).

Oxygen deficiency will occur if the blood oxygen level cannot meet the vigorous metabolic demand of tumor cells (16). Hypoxia has been proved to enhance the proliferation and angiogenesis of HCC, leading to an overall increase in tumor invasion (17). It is confirmed that the change of transverse relaxation rate of tissue R2* is linear with the concentration of deoxyhemoglobin, which can be used to detect the blood oxygen level of tumor (18). Enhanced T2*-weighted angiography (ESWAN) combines a unique 3D T2* based on multi-echo acquisition with a special reconstruction algorithm to obtain phase, magnitude, T2*, and R2* maps. ESWAN not only visualizes and delineates small vessels and microbleeds, but also helps to observe the physiological and pathological conditions (19). Automatically quantitative intratumoral susceptibility signal (ITSS) which is generated from phase image of ESWAN can reflect the neovascularization and hemorrhage in tumor (20) and the tumor angiogenesis can also directly lead to the occurrence of MVI (21). Studies have showed the effectiveness of ITSS in the assessment of intratumoral vascularity in HCC using subjective and objective semi-quantitative methods (22). Intravoxel incoherent motion (IVIM) applies multiple b values and biexponential models for the acquisition and analysis of images, which can distinguish information regarding the pure tissue diffusion of water molecules and microcirculation perfusion without the use of contrast agent (23), reflecting the tissue density and blood supply status. The changes of microcirculation and microstructure in tumor tissue may be related to the status of MVI (24).

The purpose of this study was to investigate the value of the combined application of ESWAN (R2*+ITSS) and IVIM (D+D*+f) for prediction of MVI in patients with HCC, which may help clinicans choose appropriate surgical procedures based on risk-benefit assessment.

## 2 Materials and methods

### 2.1 Patients

**Figure 1:**
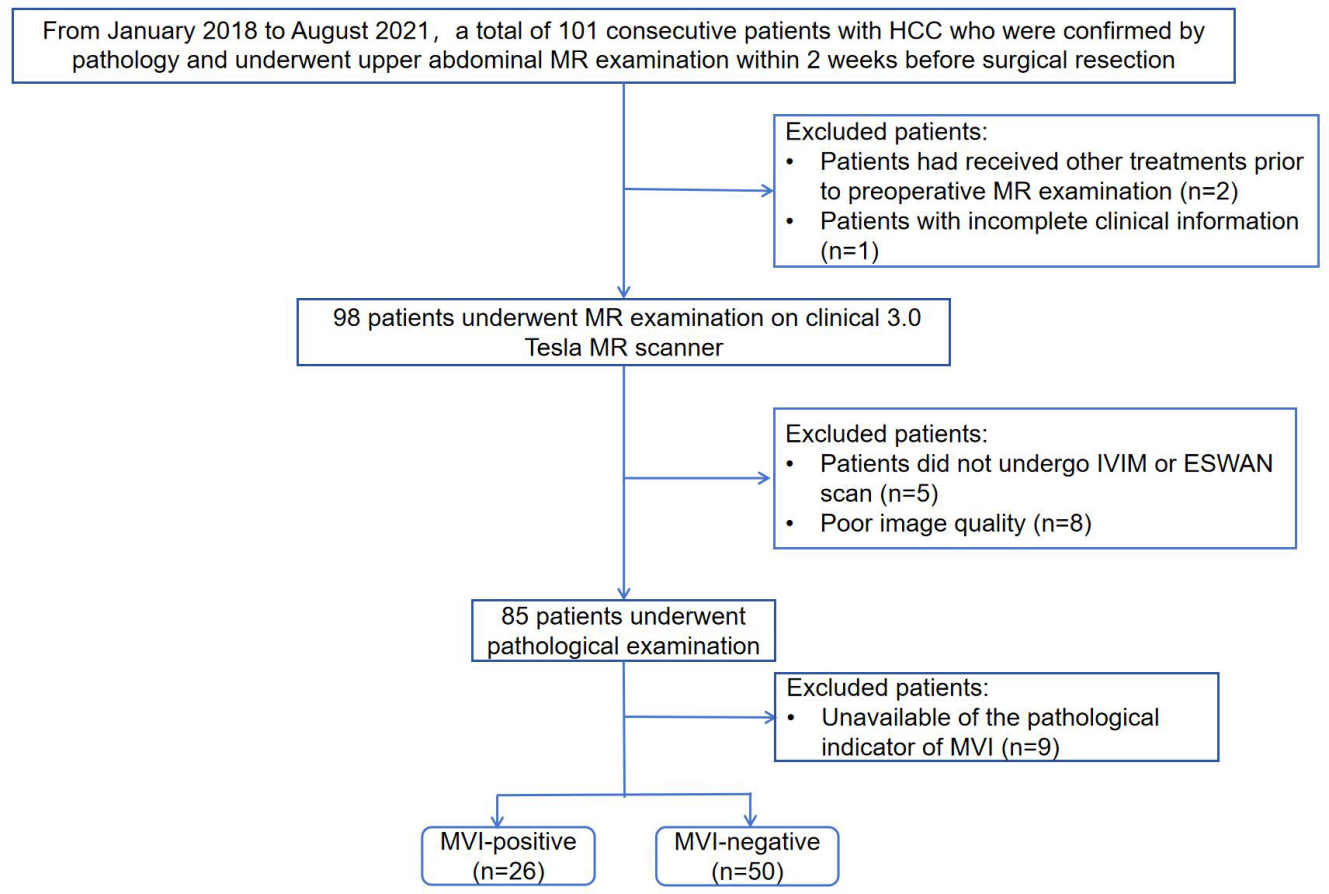
Flowchart of the study population.

This retrospective study was approved by the Institutional Review Board of our hospital and the requirement for informed consent was waived because of the retrospective nature of the study. From January 2018 to August 2021, a total of 101 consecutive patients with HCC who underwent upper abdominal MR examination were retrospectively collected. Patients meeting the following criteria were included: ①patients with pathologically confirmed HCC after surgical resection; ②patients underwent abdominal MR examination on 3.0 Tesla MR scanner within 2 weeks before resection. The exclusion criteria were as follows: ①patients had received other treatments prior to preoperative MR examination such as transarterial chemoembolization (TACE), radiofrequency ablation (RFA) or chemotherapy (n=2); ②patients with incomplete clinical information (n=1); ③patients with incomplete IVIM or ESWAN scan (n=5); ④poor image quality (n=8); ⑤unavailable of the pathological indicator of MVI (n=9). Finally, a total of 76 patients were included in the study. Flowchart of the study population is shown in **Figure 1**.

Baseline clinical information, including age, gender, history of hepatitis, alanine aminotransferase level (ALT), aspartate aminotransferase (AST), γ-Glutamyltransferase (GGT), total bilirubin (TBIL), alpha fetoprotein (AFP), carcinoembryonic antigen (CEA), and Child-Pugh class, within one week before surgery were collected.

### 2.2 Histopathological examination

Hepatic specimens resected by surgery were used for the hispathological evaluation. All hispathological examinations were performed by a team of pathologists, each individual with more than 10 years of experience in evaluating histopathological slices. The pathologists who were blinded to all clinical and imaging results reviewed the H and E-stained slides and confirmed the hispathological diagnosis. MVI was defined as the tumor within a vascular space lined by endotheliumon miscroscopy. The MVI grades were recorded by the following criteria: ①M0: no MVI; ②M1 or low risk: MVI < 5 vessels and ≤ 1 cm away from the adjacent peritumoral liver tissue; and ③M2 or high risk: MVI > 5 vessels or > 1 cm away from the adjacent peritumoral liver tissue (5). M1 and M2 were classified as MVI-positive group and M0 as MVI-negative group.

### 2.3 MR imaging

All patients performed MR examnination using a 3.0T MR scanner (Signa Excite HDxt, General Electric Healthcare, Milwaukee, WI, USA) with an 8-channel phased-array body coil. The patients were fasted for 4–6 hours and trained to exhale and hold their breath for more than 20 seconds before scanning. The patients were examined in the supine position. Conventional MR scanning sequences included fast spoiled dual-echo T1-weighted gradient-recalled-echo imaging (in phase and opposed-phase), fat-suppressed fast spin-echo T2-weighted imaging (T2WI), and liver acceleration volume acquisition (LAVA) acquired with gradient recalled echo sequence at arterial phase (40 s), portal venous phase (70 s), and delayed phase (90 s). The IVIM sequence was collected under free breathing, and a total of 12 b-values were selected (0, 20, 50, 100, 150, 200, 400, 800, 1200, 2000, 3000, and 4000 s/mm^2^). The ESWAN sequence was acquired using a 3D-enhanced T2* susceptibility-weighted angiography with contrast flow compensated multi-echo gradient echo sequence. The detailed parameters of each acquisition sequence are shown in **Table 1**.

**Table 1:** The detailed parameters of each acquisition sequence.

Sequences	Scan time (s)	Number of excitation (NEX)	Repetition time (ms)	Echo time (ms)	Field of view (mm^2^)	Slice thicknes (mm)	Slice gap (mm)	Scan matrix
Dual-echo T1-weighted gradient-recalled-echo imaging	32	1	4	2.5/1.2	375×305	6.0	1.0	208×169
T2WI	135	1	1500	117	360×360	6.0	1.0	276×276
Contrast-enhanced imaging	64	1	3.1	1.06	400×341	2.0	1.0	224×190
IVIM	189	1	2241	95	380×323	8.0	1.0	128×105
ESWAN	18	1	31	7.2	300×418	6.0	1.0	152×211

### 2.4 Radiological features evaluation

The imaging features were analyzed independently by two experienced radiologists (reviewers 1 and 2, X.R. and Y.Z., with 3 and 8 years of experience in abdominal MRI diagnosis, respectively) who were blinded to the clinical data, imaging reports, and pathological findings. The consensus was achieved by discussion when there was a disagreement between two radiologists.

The two experienced radiologists recorded the following features: ①tumor number: single or multinodular; ②tumor size, defined as the largest diameter of the tumor; ③tumor margin: defined as smooth or irregular margin; ④tumor capsule: defined as a rim of increasing enhancement surrounding the tumor in the delayed phase; ⑤enhancement pattern: typical (arterial phase washin and portal venous or delayed phase washout) or atypical enhancement pattern (hypovascular lesions which only showed washin or washout); ⑥peritumoral enhancement: the tumor border with detectable enhancement in the arterial phase and becoming isointensity in the portal venous or delayed phase compared with the background liver parenchyma (25).

### 2.5 Imaging analysis

**Figure 2:**
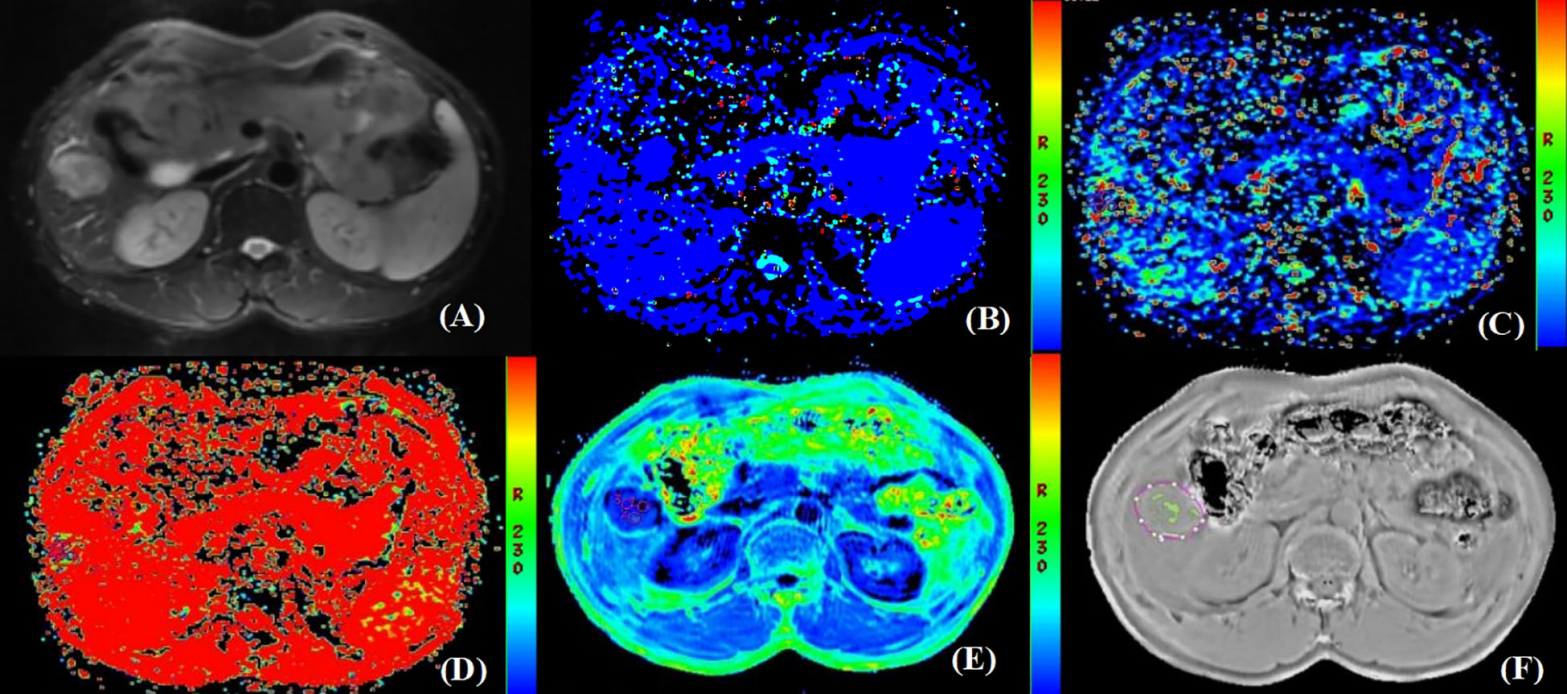
Pathologically confirmed HCC with MVI in a 66-year-old male. **(A)** T_2_W image. The circular lesion in the hepatic right lobe presented with a slightly high signal intensity and a clear boundary. **(B–E)** Parametric maps (D, D*, f, and R2*, respectively). The calculated average values of D, D*, f, and R2* for the drawn ROIs were 0.402×10^3^mm^2^/s, 0.032mm^2^/s, 0.435×100%, and 45.482Hz, respectively. **(F)** The ROIs were delineated along the tumor edge and the ITSS-ratio was automatically calculated which showed as the green area within the tumor. The ITSS-ratio was 0.200.

**Figure 3:**
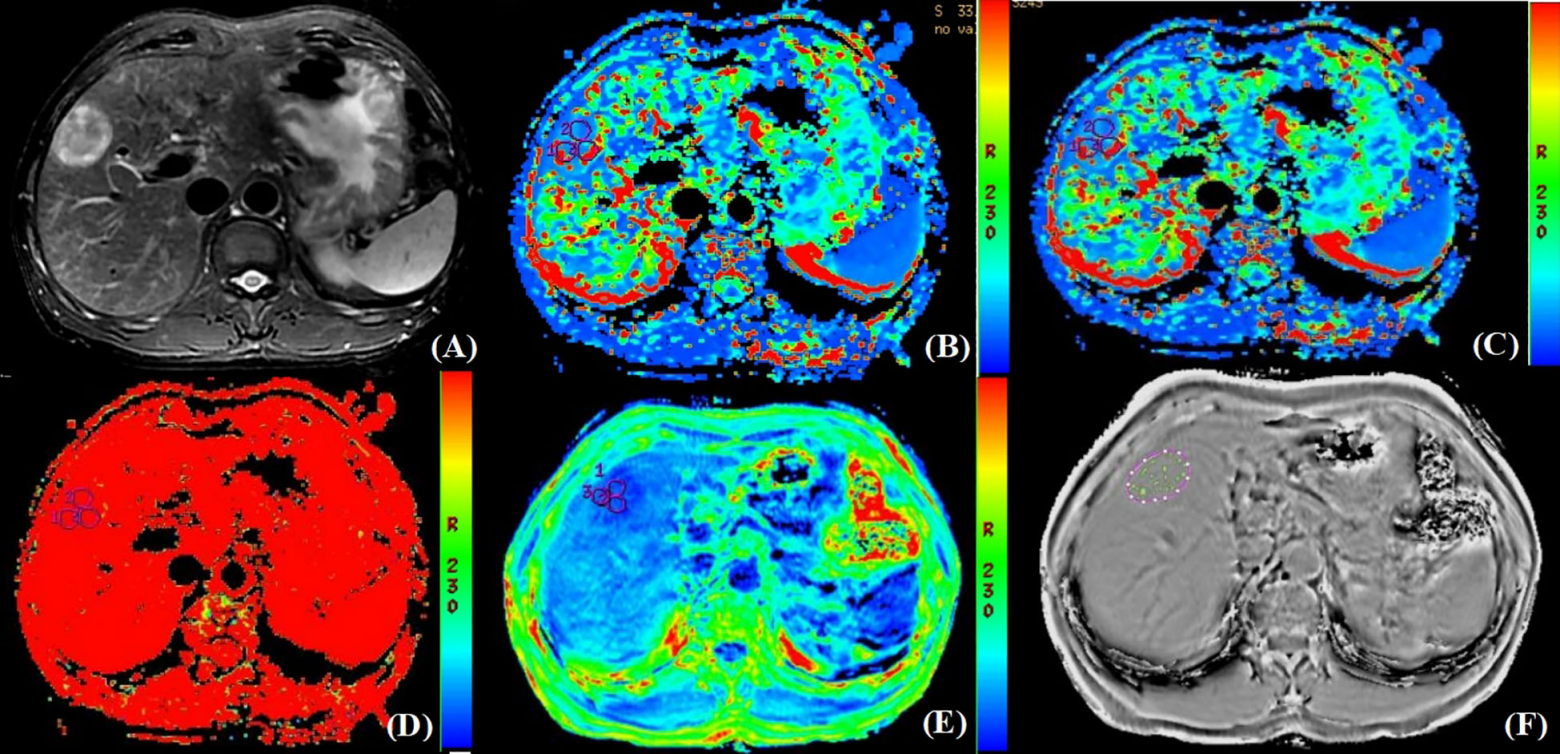
Pathologically confirmed HCC without MVI in a 62-year-old male. **(A)** T_2_W image. The circular lesion in the hepatic right lobe presented with a high signal intensity and a clear boundary. **(B–E)** Parametric maps (D, D*, f, and R2*, respectively). The calculated average values of D, D*, f, and R2* for the drawn ROIs were 1.480×10^3^mm^2^/s, 0.130 mm^2^/s, 0.471×100%, and 25.625Hz, respectively. **(F)** The ROIs were delineated along the tumor edge and the ITSS-ratio was automatically calculated which showed as the green area within the tumor. The ITSS-ratio was 0.031.

The IVIM and ESWAN raw data were transferred to the AW 4.6 workstation (GE Healthcare). The Functool software was used to generate IVIM and ESWAN parametric maps and quantify corresponding parameters. The parameters were measured independently by two radiologists (reviewers 1 and 2, X.R.and Y.Z.) who were blinded to clinical and pathological information of the patients. Referring to the lesion location information obtained on T_2_W images, the radiologists manually placed three region of interests (ROIs) (100 - 200 mm^2^) on the largest slice of the lesion (26) on D, D*, and f maps derived from IVIM sequence, and R2* map derived from ESWAN sequence (**Figures 2**, **3**). The ROIs were required to avoid the hemorrhage, calcify, and necrotic areas. For HCC patients with multiple lesions, the largest lesion was selected for analysis (27). The average value of each parameter was used for statistical analysis.

Furthermore, the ITSS was automatically calculated using the Anatomy Sketch (A.S.) software (Dalian University of Technology). Firstly, we removed the artifacts of the phase map obtained by processing ESWAN sequence. Secondly, the phase map in NII format after artifact removal was imported into A.S. software, the lesion was identified by two radiologists according to the axial T_2_W image by double-blind method, and then three ROIs were delineated along the tumor edge in the first layer, the last layer and any layer between them on the phase map. Finally, the A.S. software was used to automatically calculate the ITSS ratio of the largest layer of the tumor (**Figures 2**, **3**).

### 2.6 Statistical analysis

The SPSS 27.0 software (SPSS Inc., Chicago, IL, USA) was used for statistical analyses. For categorical variables, the interobserver agreement was assessed by using the kappa test. The agreement was interpreted as a slight, fair, moderate, substantial, and almost perfect agreement when the estimated kappa values were 0–0.20, 0.21–0.40, 0.41–0.60, 0.61–0.80, and over 0.80, respectively. Categorical variables were recorded as the number of cases and percentages, and χ2 or Fisher’s exact test was used to compare the difference between MVI-positive and MVI-negative groups.

For continuous variables, the intraclass correlation coefficient (ICC) was used to evaluate the consistency between the two observers (ICC value < 0.4 indicated poor consistency, ICC value > 0.75 indicated good consistency). The normality of the variables was tested by the Shapiro-Wilk test. Normally distributed data were expressed as means ± standard deviations, and non-normally distributed data were expressed as medians and ranges (25th, 75th percentiles). The significance of the inter-group difference was determined using the independent sample t-test or Mann-Whitney U-test for normally or non-normally distributed data. Univariate and multivariate logistic regression analyses were used to screen the independent risk factors of MVI. Logistic regression analysis was used to generate the predicted probability of MVI for the IVIM, ESWAN, and the combination of IVIM and ESWAN parameters. Receiver operating characteristic (ROC) curves were drawn to evaluate the ability for predicting MVI. The cutoff point was selected by using the maximized value of Youden index, and sensitivity and specificity at the threshold value were determined. The Delong’s test was used to compare the differences between the AUCs. A *P* value less than 0.05 was considered to indicate a statistical significance.

## 3 Results

### 3.1 Clinical and imaging characteristics

This study included 76 HCC lesions from 76 patients (57 males; mean age, 60.16 ± 9.05 years; range, 31–80 years). According to the histopathological reports, 26 patients were MVI-positive (17 cases were classified as M1 and 9 cases were classified as M2) and 50 patients were MVI-negative (50 cases were classified as M0). Among 76 patients, 9 cases were multiple HCC. Clinical and imaging characteristics of all patients are summarized in **Table 2**. The ALT (*P*=0.009), AST (*P*=0.039), AFP (*P*=0.003), tumor size (*P*=0.008), and peritumoral enhancement (*P*=0.022) showed statistically significant differences between the MVI-positive and MVI-negative groups. After univariate and multivariate analyses, only AFP (odds ratio, 0.183; 95% CI, 0.041–0.823; *P* = 0.027) was the independent risk factor for predicting the status of MVI (**Table 3**). The AUC of AFP for prediction of MVI were 0.652, with a sensitivity of 92.0% and a specificity of 38.5% (cut-off value: 1.500).

**Table 2:** Clinical and imaging characteristics of patients.

Variables	MVI-negative (n=50)	MVI-positive (n=26)	t/Z/*Χ* ^2^	*P* value
Age^a^	59.270 ± 9.075	60.620 ± 9.098	0.615	0.541
Gender^b^			0.078	0.780
Male	37.0 (74.0)	20.0 (76.9)		
Female	13.0 (26.0)	6.0 (23.1)		
History of hepatitis^b^			1.395	0.238
Present	34.0 (68.0)	21.0 (80.8)		
Absent	16.0 (32.0)	5.0 (19.2)		
ALT^b^			6.780	0.009*
>50 U/L	9.0 (18.0)	12.0 (46.2)		
≤50 U/L	41.0 (82.0)	14.0 (53.8)		
AST^b^			4.257	0.039*
>40 U/L	10.0 (20.0)	11.0 (42.3)		
≤40 U/L	40.0 (80.0)	15.0 (57.7)		
GGT^b^			0.053	0.818
>60 IU/L	16.0 (32.0)	9.0 (34.6)		
≤60 IU/L	34.0 (68.0)	17.0 (65.4)		
TBIL^b^			0.097	0.755
>20 umol/L	10.0 (20.0)	6.0 (23.1)		
≤20 umol/L	40.0 (80.0)	20.0 (76.9)		
AFP				0.003*
≥400 ng/ml	4.0 (8.0)	10.0 (38.5)		
<400 ng/ml	46.0 (92.0)	16.0 (61.5)		
CEA				0.482
≥5 ng/ml	5.0 (10.0)	4.0 (15.4)		
<5 ng/ml	45.0 (90.0)	22.0 (84.6)		
Child-Pugh class^b^				0.114
Child-Pugh A	47.0 (94.0)	21.0 (80.8)		
Child-Pugh B	3.0 (6.0)	5.0 (19.2)		
Tumor size^a^ (cm)	3.600 (1.975, 5.225)	5.400 (3.243, 9.425)	-2.661	0.008*
Number of the tumors^b^				1.000
Single	43.0 (86.0)	23.0 (88.5)		
Multinodular	7.0 (14.0)	3.0 (11.5)		
Tumor margin^b^			0.390	0.532
Smooth margin	29.0 (58.0)	17.0 (65.4)		
Irregular margin	21.0 (42.0)	9.0 (34.6)		
Tumor capsule^b^			0.472	0.492
Present	40.0 (80.0)	19.0 (73.1)		
Absent	10.0 (20.0)	7.0 (26.9)		
Enhancement pattern^b^			1.031	0.312
Typical	35.0 (70.0)	21.0 (80.8)		
Atypical enhancement pattern	15.0 (30.0)	5.0 (19.2)		
Peritumoral enhancement^b^			5.212	0.022*
Present	9.0 (18.0)	11.0 (42.3)		
Absent	41.0 (82.0)	15.0 (57.7)		

^a^Data were compared using the independent sample t-test or Mann-Whitney U-test and expressed as mean ± standard deviation or P50 (P25, P75). ^b^Data were compared using χ2 or Fisher’s exact test and expressed as numbers with percentages. ALT, alanine aminotransferase; AST, alpha fetoprotein; GGT, γ-Glutamyltransferase; TBIL, total bilirubin; AFP, alpha fetoprotein; CEA, carcinoembryonic antigen.

The meaning of symbol “*” is that the P value less than 0.05 and is considered to indicate a statistical significance.

**Table 3:** Univariate and multivariate analyses of clinical and imaging characteristics for predicting MVI status.

Risk factors	Univariate analysis			Multivariate analysis		
	Odds ratio	95% CI	*P*	Odds ratio	95% CI	*P*
Age	0.984	0.933-1.037	0.536			
Gender	0.854	0.281-0.259	0.780			
History of hepatitis	1.976	0.631-6.193	0.242			
ALT	0.256	0.089-0.736	0.011	0.202	0.034-1.194	0.078
AST	0.341	0.120-0.966	0.043	1.208	0.203-7.175	0.835
GGT	0.889	0.326-2.423	0.818			
TBIL	0.833	0.265-0.620	0.755			
AFP	0.139	0.038-0.506	0.003	0.183	0.041-0.823	0.027
CEA	0.611	0.149-2.504	0.494			
Child-Pugh class	3.730	0.815-17.072	0.090			
Tumor size	1.291	1.079-1.543	0.005	1.161	0.943-1.428	0.159
Number of the tumors	0.512	0.098-2.663	0.426			
Tumor margin	0.731	0.273-1.956	0.533			
Tumor capsule	1.474	0.486-4.470	0.493			
Enhancement pattern	0.556	0.176-1.750	0.315			
Peritumoral enhancement	0.299	0.104-0.865	0.026	0.292	0.083-1.033	0.056

### 3.2 Interobserver agreement analysis of continuous and categorical variables

As shown in **Table 4**, the agreements of categorical variables evaluated by the two observers were all excellent (kappa>0.80) and the agreements of continuous variables between the two observers were all in good (ICC>0.75).

**Table 4:** The agreement analysis of continuous and categorical variables between the two observers.

	Observer 1		Observer 2		ICC/ Kappa
	MVI-negative	MVI-positive	MVI-negative	MVI-positive	
D (×10^-3^mm^2^/s)	0.892 (0.760, 1.303)	0.591 (0.372, 0.824)	0.818 (0.605, 1.147)	0.674 (0.391, 0.966)	0.788
D* (mm^2^/s)	0.055 (0.025, 0.100)	0.028 (0.006, 0.050)	0.054 (0.189, 0.861)	0.032 (0.006, 0.050)	0.907
f (100%)	0.431 ± 0.154	0.466 ± 0.101	0.429 (0.343, 0.594)	0.529 (0.436, 0.609)	0.778
R2* (Hz)	29.290 (23.117, 35.228)	43.696 (34.914, 58.083)	30.296 (22.871, 38.202)	40.641 (26.627, 61.354)	0.908
ITSS	0.146 (0.086, 0.236)	0.199 (0.155, 0.245)	0.180 (0.107, 0.254)	0.207 (0.162, 0.240)	0.843
Tumor size (cm)	3.600 (1.975, 5.225)	5.400 (3.243, 9.425)	3.520 (1.878, 5.158)	5.445 (3.128, 9.190)	0.993
Number of the tumors					1.000
Single	43.0 (86.0)	23.0 (88.5)	43.0 (86.0)	23.0 (88.5)	
Multinodular	7.0 (14.0)	3.0 (11.5)	7.0 (14.0)	3.0 (11.5)	
Tumor margin					0.890
Smooth margin	31.0 (62.0)	17.0 (65.4)	28.0 (56.0)	16.0 (61.5)	
Irregular margin	19.0 (38.0)	9.0 (34.6)	22.0 (44.0)	10.0 (38.5)	
Tumor capsule					0.963
Present	40.0 (80.0)	19.0 (73.1)	40.0 (80.0)	18.0 (69.2)	
Absent	10.0 (20.0)	7.0 (26.9)	10.0 (20.0)	8.0 (30.8)	
Enhancement pattern					0.809
Typical	35.0 (70.0)	21.0 (80.8)	33.0 (66.0)	19.0 (73.1)	
Atypical enhancement pattern	15.0 (30.0)	5.0 (19.2)	17.0 (34.0)	7.0 (26.9)	
Peritumoral enhancement					0.893
Present	9.0 (18.0)	11.0 (42.3)	6.0 (12.0)	11.0 (42.3)	
Absent	41.0 (82.0)	15.0 (57.7)	44.0 (88.0)	15.0 (57.7)	

### 3.3 Comparisons of IVIM and ESWAN parameters between the two groups

As shown in **Table 5**, there were statistically significant differences in D (*P*<0.001), D* (*P*=0.003), R2* (*P*<0.001), and ITSS (*P*=0.025) between MVI-positive and MVI-negative groups. The D and D* values of MVI-negative group were significantly higher than those of MVI-positive group, which in MVI-negative group were 0.892×10^-3^ (0.760×10^-3^, 1.303×10^-3^) mm^2^/s and 0.055 (0.025, 0.100) mm^2^/s, and in MVI-positive group were 0.591×10^-3^ (0.372×10^-3^, 0.824×10^-3^) mm^2^/s and 0.028 (0.006, 0.050) mm^2^/s, respectively. The R2* and ITSS values of MVI-negative group were significantly lower than those of MVI-positive group, which in MVI-negative group were 29.290 (23.117, 35.228) Hz and 0.146 (0.086, 0.236), and in MVI-positive group were 43.696 (34.914, 58.083) Hz and 0.199 (0.155, 0.245), respectively. No statistical significance was observed for f (*P*=0.239) value in those patients with MVI-positive group compared with MVI-negative group (**Figures 2**, **3**).

**Table 5:** Comparisons of IVIM and ESWAN parameters between MVI-positive and MVI-negative groups.

	MVI-negative (n =50)	MVI-positive (n = 26)	*t/Z*	*P*
D (×10^-3^ mm^2^/s)	0.892 (0.760, 1.303)	0.591 (0.372, 0.824)	-3.405	<0.001*
D* (mm^2^/s)	0.055 (0.025, 0.100)	0.028 (0.006, 0.050)	-2.945	0.003*
f (100%)	0.431 ± 0.154	0.466 ± 0.101	-1.188	0.239
R2* (Hz)	29.290 (23.117, 35.228)	43.696 (34.914, 58.083)	-4.248	<0.001*
ITSS	0.146 (0.086, 0.236)	0.199 (0.155, 0.245)	-2.239	0.025*

The meaning of symbol “*” is that the P value less than 0.05 and is considered to indicate a statistical significance.

**Figure 4:**
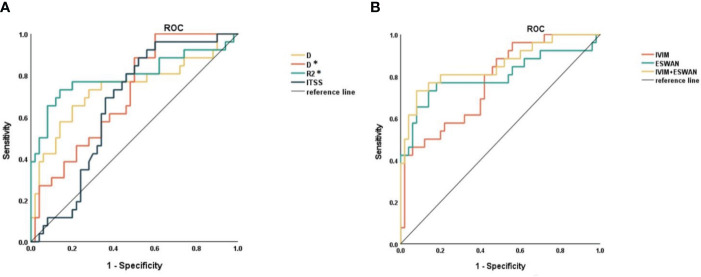
**(A)** ROC curves of D, D*, R2*, and ITSS for distinguishing MVI-positive and MVI-negative in HCC. **(B)** ROC curves of IVIM, ESWAN, and combination for distinguishing MVI-positive and MVI-negative in HCC.

The AUCs of D, D*, R2*, and ITSS for prediction of MVI were 0.739, 0.707, 0.798, and 0.657, respectively (**Table 6**, **Figure 4**). The AUCs of IVIM (D+D*), ESWAN (R2*+ITSS), and combination (D+D*+R2*+ITSS) were 0.772, 0.800, and 0.855, respectively (**Table 5**, **Figure 4**). When IVIM combined with ESWAN, the performance was improved with a sensitivity of 73.1% and a specificity of 92.0% (cut-off value: 0.502) and was significantly higher than AFP (*P*=0.001), D (*P*=0.038), D* (*P*=0.023), R2* (*P*=0.034), and ITSS (*P*=0.005), respectively.

**Table 6:** Predictive performance of clinical and imaging characteristic, IVIM and ESWAN parameters between MVI-positive and MVI-negative groups.

Parameters	AUC	Cut-off value	Sensitivity (%)	Specificity (%)	Compared to combination (IVIM+ESWAN)	
					*Z*	*P*
Clinical and imaging characteristic (AFP)	0.652	1.500	92.0	38.5	3.222	0.001*
D (mm^2^/s)	0.739	0.727 × 10^-3^	80.0	65.4	2.076	0.038*
D* (mm^2^/s)	0.707	0.068	40.0	100.0	2.271	0.023*
R2* (Hz)	0.798	39.186	86.0	73.1	2.120	0.034*
ITSS	0.657	0.136	48.0	88.5	2.806	0.005*
IVIM (D+D*)	0.772	0.249	88.5	52.0	1.731	0.084
ESWAN (R2*+ITSS)	0.800	0.416	73.1	86.0	1.870	0.061
IVIM+ESWAN (D+D*+R2*+ITSS)	0.855	0.502	73.1	92.0		

The meaning of symbol “*” is that the P value less than 0.05 and is considered to indicate a statistical significance.

## 4 Discussion

The major finding of this work was that both IVIM and ESWAN quantitative parameters could be used to preoperatively predict MVI status of HCC. We found that D and D* values were significantly decreased, while R2* and ITSS values were significantly increased with the presence of MVI in HCC. And the combination of IVIM and ESWAN parameters showed better discriminative efficacy in comparison with clinical and imaging characteristics, individual IVIM/ESWAN parameter or sequence separately for prediction of MVI in patients with HCC. Our finding may be useful to provide optimized treatment and achieve long-term survival for patients with HCC.

Some researchers reported that D value derived from IVIM was an independent risk factor for predicting MVI in HCC (24, 25), but there is a certain limitation in the number of patients and the predictive performances need to be improved. In our study, both D and D* in HCC with the presence of MVI were significantly lower than those in HCC patients without MVI. This maybe because that the presence of MVI increases the infiltration of tumor cells, provides more nutrients for tumor cell proliferation, and makes the tumor cell structure more dense, thus limiting the diffusion of water molecules (24), resulting in decreased D value. MVI may cause decreased perfusion through occlusion of microvessels, thus resulting in decreased D* value.

R2* value, the transverse relaxation rate, is quantitative index to evaluate the change of oxygen contents in local tissues, and is related to hypoxia, bleeding, abnormal blood vessels and other factors. Deoxyhemoglobin, metallohemoglobin, hemoglobin, ferritin, and calcification, as magnetically sensitive materials, can produce small magnetic fields. These materials result in an inhomogeneity of the local field, change the phase of the proton’s spin, and accelerate the decay of the signal intensity; thus, the R2* value increases (28). ESWAN is extremely sensitive to paramagnetic deoxyhemoglobin and hemosiderin, so oxygenation levels in the tissues can be detected by measuring the R2* value (29). Chen et al. (30) found that ESWAN was better at identifying certain morphologic features such as pseudocapsule and hemorrhage than conventional MRI without using a contrast agent in HCC patients. Studies have showed that the sensitivity and specificity of R2* in distinguishing HCC from hepatic cavernous hemangioma were 96.20% and 97.80% and the AUC for the diagnosis of HCC is 0.994, respectively (31). By quantitative analysis of R2* value obtained by post-processing post oxygen (O_2_) breathing BOLD MRI, Kartiketal et al. (32) found that the R2* value between the groups with and without MVI was not significantly different statistically. Since then, no relevant studies using R2* value to predict MVI status of HCC have been reported. Our study showed that R2* was significantly higher in the MVI-positive group than that in the MVI -negative group. This may be due to more malformed neovascularization and higher metabolism in the MVI-positive group, leading to tumor being in a state of relative oxygen deficiency. The reasons for the difference from the previous results are as follows: BOLD MRI analysis is more susceptible to the stimulation intensity and stimulation time due to its imaging method, which lead to the lack of stability and reliability of the quantitative analysis results of BOLD imaging. Secondly, the higher field intensity (3.0T) used in our study will enlarge the R2* quantitative spectrum and may be more sensitive to the difference of oxygen content.

ITSS are low signal areas in the tumor that presents as continuous dots or thin lines on the phase map. Probably due to angiogenesis and increased blood supply, the tumor contains a relatively large amount of deoxyhemoglobin, which generates susceptibility effects and causes signal-intensity loss (33). Yang et al. (22) demonstrated that ITSS could provide a new biomarker for evaluating the antiangiogenic effect of sorafenib in HCC. At present, the evaluation of ITSS is mainly based on its size and frequency, but manual evaluation is time-consuming, laborious and subjective. Bhattacharjee et al. (34) used MATLAB software to quantify ITSS for predicting MVI in hepatitis B virus-related combined HCC and cholangiocarcinoma, which can quantify the volume of ITSS in the whole tumor, but it is only suitable for tumors with ITSS visible to the naked eye, and the image has not been artifact-removed, which may lead to inaccurate results. In our study, automatic extraction and quantitative ITSS are used for the first time to predict MVI of HCC, which is simple and easy to operate, and there is no need to pre-screen the images of ITSS visible to the naked eye. In addition, phase map is used to remove artifacts, which is more suitable for abdominal organs prone to motion artifacts. The result of our study showed that the ITSS of MVI-positive group was significantly higher than that of the MVI-negative group, suggesting a higher microvascular density with the presence of MVI. This is consistent with the pathological process of MVI. The irregular course of tumor blood vessels increases blood flow resistance, prolongs blood retention time, overuses blood oxygen, and leads the increase of deoxyhemoglobin and its oxidized products. Ultimately, the ITSS increases.

In our study, only AFP was the independent risk factor in clinical characteristics for predicting the status of MVI, which was consistent with the previous study (35), suggesting that high levels of AFP can respond to tumor cell invasion (36). The AUC of AFP for prediction of MVI was 0.652, and was significantly lower than the combination of IVIM and ESWAN (P=0.001).

Our study has several limitations. Firstly, this was a retrospective study and the sample size was relatively small. We will collect more cases prospectively from our center or other centers to validate the reliability of our results in the following research. Secondly, we did not evaluate MVI on a per-lesion basis but on a per-patient basis. We are unable to correlate the ROI we plotted on MR images with site-by-site pathological vascular invasion. Thirdly, measurements were not obtained from the entire volume of tumors, but in its largest dimension, so part of information about the tumor’s heterogeneity may have been missed. The whole tumor will be measured in the future research. Fourthly, previous studies (37, 38) have shown that the imaging features around the tumor are good predictors for MVI status, we will perform EAWAN and IVIM quantitative analysis of peritumoral areas in the follow-up study.

In our study, we combined the quantitative parameters of IVIM and ESWAN sequences and automatic extraction and quantification of ITSS to analyze the perfusion and oxygenation levels in HCC with or without MVI. The IVIM and ESWAN parameters showed good efficacy in prediction of MVI in patients with HCC. The combination of IVIM and ESWAN may be useful for noninvasive prediction of MVI before clinical operation, which may potentially aid in improving the prediction of HCC patients’ survival and preventing recurrence and metastasis.

## Data Availability

The raw data supporting the conclusions of this article will be made available by the authors, without undue reservation.
